# Boom boom pow: Shock-facilitated aqueous alteration and evidence for two shock events in the Martian nakhlite meteorites

**DOI:** 10.1126/sciadv.aaw5549

**Published:** 2019-09-04

**Authors:** L. Daly, M. R. Lee, S. Piazolo, S. Griffin, M. Bazargan, F. Campanale, P. Chung, B. E. Cohen, A. E. Pickersgill, L. J. Hallis, P. W. Trimby, R. Baumgartner, L. V. Forman, G. K. Benedix

**Affiliations:** 1 School of Geographical and Earth Sciences, University of Glasgow, Glasgow G12 8QQ, UK.; 2 Space Science and Technology Centre, School of Earth and Planetary Sciences, Curtin University, GPO Box U1987, Perth, WA 6845, Australia.; 3 Australian Centre for Microscopy and Microanalysis, The University of Sydney, NSW 2006, Australia.; 4 School of Earth and Environment, University of Leeds, Leeds LS2 9JT, UK.; 5 Department of Earth Sciences, Uppsala University, Uppsala, Sweden.; 6 Dipartimento di Scienze della Terra, Università di Pisa, via Santa Maria 53, 56126, Pisa, Italy.; 7 Oxford Instruments Nanoanalysis, High Wycombe HP12 3SE, UK.; 8 Australian Centre for Astrobiology, University of New South Wales, Sydney, NSW 2052, Australia.; 9 Department of Earth and Planetary Sciences, Western Australia Museum, Locked Bag 49, Welshpool, WA 6986, Australia.; 10 Planetary Science Institute, 1700 East Fort Lowell, Suite 106, Tucson, AZ 85719-2395, USA.

## Abstract

Nakhlite meteorites are ~1.4 to 1.3 Ga old igneous rocks, aqueously altered on Mars ~630 Ma ago. We test the theory that water-rock interaction was impact driven. Electron backscatter diffraction demonstrates that the meteorites Miller Range 03346 and Lafayette were heterogeneously deformed, leading to localized regions of brecciation, plastic deformation, and mechanical twinning of augite. Numerical modeling shows that the pattern of deformation is consistent with shock-generated compressive and tensile stresses. Mesostasis within shocked areas was aqueously altered to phyllosilicates, carbonates, and oxides, suggesting a genetic link between the two processes. We propose that an impact ~630 Ma ago simultaneously deformed the nakhlite parent rocks and generated liquid water by melting of permafrost. Ensuing water-rock interaction focused on shocked mesostasis with a high density of reactive sites. The nakhlite source location must have two spatially correlated craters, one ~630 Ma old and another, ejecting the meteorites, ~11 Ma ago.

## Teaser

Evidence for impact-generated water on Mars ~633 Ma ago predicts two craters at the nakhlite meteorite’s ejection site.

## INTRODUCTION

The nakhlite meteorites are a suite of pyroxene-rich mafic igneous rocks from Mars (*1*). They typically comprise elongate, subhedral to euhedral prisms of augite (68 to 81 volume %), phenocrysts of olivine (3 to 17 volume %), and an interstitial fine-grained mesostasis (8 to 22 volume %) (*1*). A distinct foliation and lineation petrofabric are defined by the pyroxene phenocrysts (*2*–*4*). Crystallization ages of the nakhlites span 93 Ma, from ~1416 to 1322 Ma ago, and indicate that they were emplaced in at least four magmatic events (*5*). Mild aqueous alteration of the nakhlites is evidenced by a suite of predominantly hydrous minerals, collectively termed “iddingsite” (*2*). These alteration products occur mainly as crystallographically controlled veins within olivine phenocrysts and less commonly as veins and irregular patches within the mesostasis (*1*, *2*). Water-rock interaction took place at ~633 ± 23 Ma ago (*6*); i.e., ~700 Ma after the nakhlite’s magmatic origin. The hydrogen isotopic composition of iddingsite is consistent with derivation of the water from atmospheric or crustal reservoirs (*7*).

The nakhlites have microstructures that are characteristic of mild shock metamorphism, <15 GPa (*1*), which has been interpreted to record their ejection from Mars’ surface by an impact ~11 Ma ago (*1*). These microstructures include undulatory extinction in olivine and pyroxene phenocrysts, as well as microfaults/fractures and sparsely distributed brecciated regions within which pyroxene phenocrysts have abundant (001)-parallel mechanical twins. The phase maskelynite (used here to denote a shock-induced glass with the composition of plagioclase feldspar), which forms by high-pressure (>15 GPa) shock, has not been reported (*1*, *3*, *8*, *9*).

The driver of aqueous alteration of the nakhlites is unknown yet could provide unique insights into the processes that were capable of generating liquid water within the Amazonian crust of Mars. Because the alteration products formed ~700 Ma after nakhlite magmatism and ~600 Ma before the ejection impact, neither process could have driven water-rock interaction (*6*, *10*). Here, we ask whether the nakhlites preserve evidence for an additional impact that could have been responsible for aqueous alteration. We have sought petrographic evidence for a shock event concurrent with aqueous alteration ~630 Ma ago by analyzing two nakhlites using scanning electron microscope (SEM) techniques, including large-area electron backscatter diffraction (EBSD) mapping coupled with computational modeling. We used thin sections of Lafayette (USNM 1505-5) and Miller Range (MIL) 03346 (MIL 03346,118). These meteorites were selected because they originated from different parts of the nakhlite lava sequence, as evidenced by their contrasting ^40^Ar/^39^Ar ages [1321.7 ± 9.6 Ma old and 1390.9 ± 8.9 Ma old, respectively (*5*)]. They also have distinct petrographic characteristics (*11*, *12*) and so may have responded differently to the passage of a shock wave.

### Petrography of Lafayette and MIL 03346

Lafayette has a modal mineralogy of 75.5 volume % augite, 12.8 volume % olivine, 6.8 volume % mesostasis, 3.6 volume % iddingsite, 1.3 volume % orthopyroxene, and 0.9 volume % Fe–Ti oxide (*13*). The cores of augite and olivine grains have average compositions of En_37_Fs_24_Wo_39_ and Fa_69_, respectively (*14*). Areas of mesostasis are 100 to 600 μm in size and contain plagioclase [Ab_62.6_An_31.8_Or_5.6_; (*2*)] and alkali feldspar, Fe-rich clinopyroxene (pigeonite), titanomagnetite, ilmenite, pyrite, K-Na aluminosilicate glass, and iddingsite (*15*).

The modal mineralogy of MIL 03346 is 76.9 volume % augite, 21.0 volume % mesostasis, 1.8 volume % titanomagnetite, and 0.3 volume % olivine. The augite grains have average core compositions of W_38–42_En_35–40_Fs_22–28_ and narrow hedenbergite rims where augite grain edges are in contact with the mesostasis (*16*). Coarse poikilitic olivine grains are zoned (Fo_43_ core to Fo_25_ rim) (*16*). Feldspathic glass is the main constituent of the mesostasis and occurs in three compositionally distinct varieties (Al rich, Al poor, and K-P-Ca rich) (*16*); Raman spectroscopy has shown that some of this material is poorly crystalline feldspar (*17*). MIL 03346 mesostasis also contains crystals of sulfide (mainly pyrrhotite), skeletal titanomagnetite with ilmenite lamellae, fayalite (Fa_92–96_) that has been partially altered to laihunite, apatite, hematite, and cristobalite (*16*, *18*).

## RESULTS

### Deformation microstructures and their distribution

**Fig. 1:**
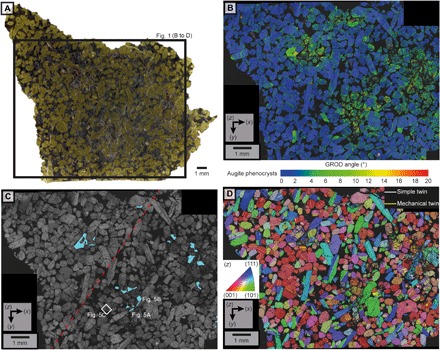
Distribution of deformation and aqueous alteration in MIL 03346. (**A**) Transmitted light image of the MIL 03346 thin section. The dominant augite phenocrysts impart the green color, whereas mesostasis is black. Areas where the mesostasis has been altered are red brown, and they correlate with the location of the deformed regions in (B). The black box indicates the area analyzed by EBSD in (B) to (D). (**B**) GROD angle EBSD map showing that deformation occurs in regions ~2 mm in size that are separated by 1- to 3-mm undeformed areas. Undeformed crystals are shown in blue, whereas increasing internal deformation is highlighted by a progression of green through yellow to red. (**C**) Band contrast EBSD map showing diminished band contrast [lower electron backscatter pattern (EBSP) quality] as darker regions that correlate with the deformed regions of (B). The blue polygons indicate the location of areas of altered mesostasis, which also correlate with deformed regions in (B). The red dashed line is the orientation of the foliation described by Daly *et al.* (*4*). The white box and labels indicate the sites where high-resolution backscattered electron (BSE) images and energy dispersive x-ray spectroscopy (EDS) images were acquired for Fig. 5 (A to C). (**D**) Inverse pole figure map highlighting the distribution of twinned augite crystals. Mechanical twins, whose boundaries are shown as yellow lines, occur exclusively within the deformed areas of (B). Simple twin boundaries are shown as white lines and occur throughout the sample. See fig. S3 for a high-resolution version of this image.

**Fig. 2:**
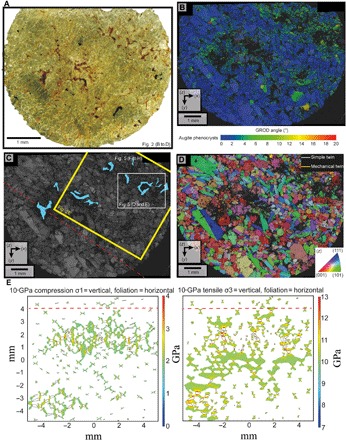
Distribution of deformation and aqueous alteration in Lafayette. (**A**) Transmitted light image of the thin section. The dominant augite phenocrysts impart the green color. Areas where the mesostasis has been altered are red brown, and they correlate with the location of the deformed regions in (B). The black box indicates the area analyzed by EBSD in (B) to (D). (**B**) GROD angle EBSD map showing that deformation occurs in regions ~2 mm in width with undeformed areas between 1 and 3 mm in size. Undeformed crystals are shown in blue, whereas increasing internal deformation is highlighted by a progression of greens through yellow to red. (**C**) Band contrast EBSD map showing diminished band contrast (lower EBSP quality) as darker regions that correlate with the deformed regions of (A). The blue polygons indicate the location of altered mesostasis, which correlates with deformed regions in (B). The red dashed line is the orientation of the foliation described by Daly *et al.* (*4*). The white box and labels indicate the sites where high-resolution BSE images and EDS images acquired for Fig. 5 (D to H). The yellow box indicates the region of Lafayette modeled in the numerical simulation in (E). (**D**) Inverse pole figure map highlighting the distribution of twinned augite. Mechanical twins, whose boundaries are shown as yellow lines, occur exclusively in the vicinity of the deformed regions of (B). Simple twin boundaries are shown as white lines and occur throughout the sample. See fig. S4 for a high-resolution version of this image. (**E**) Results from numerical simulation of the local pressure distribution from the passage of a 10-GPa horizontal compressive (left) and horizontal tensile (right) shock wave. The modeled area of Lafayette highlighted in (C). High-pressure spikes are associated with regions of high mesostasis abundance (black polygons); high-pressure loci are associated with mesostasis-phenocryst contacts. The pattern of deformation mirrors the foliation (red dashed line) and regions of abundant shock-related deformation [high GROD, fracturing, and (100) twinning] in Lafayette.

**Fig. 3:**
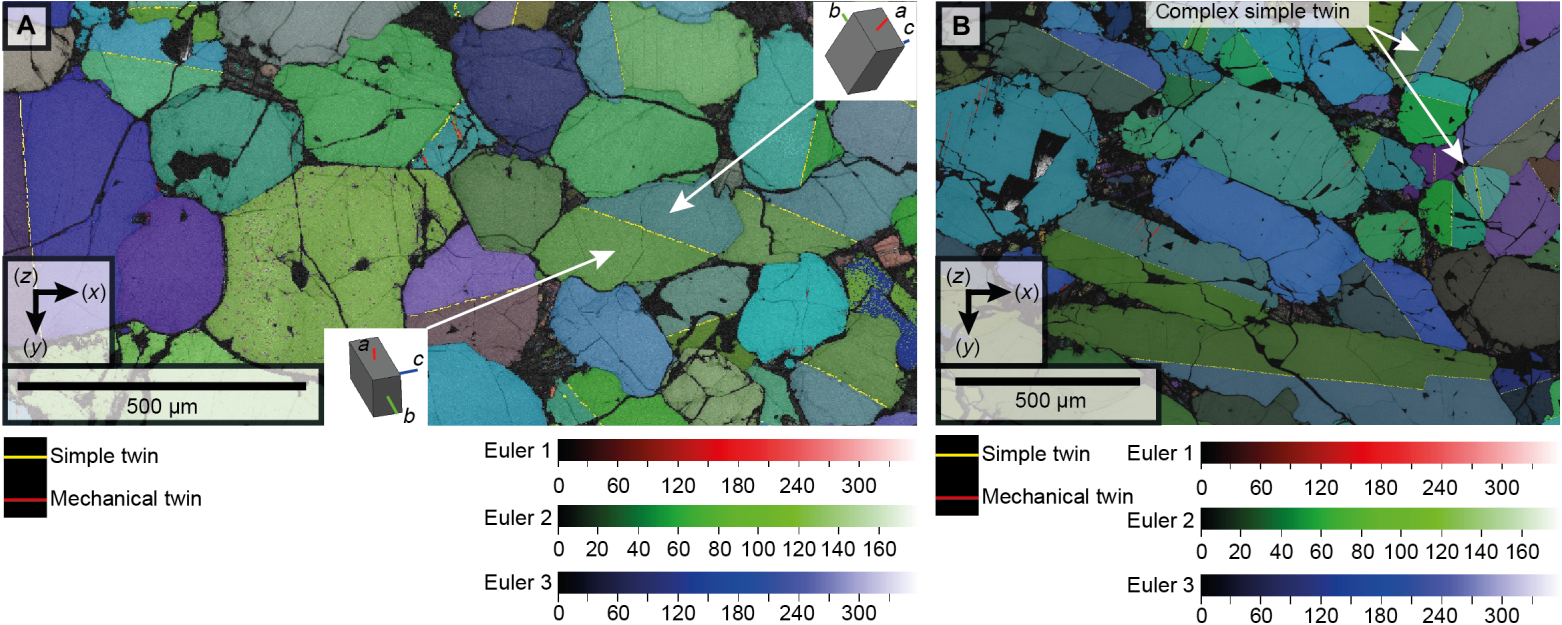
Microstructures of undeformed areas exhibiting simple twinning and no lattice bending. High-resolution (0.5- to 1.5-μm step size) EBSD Euler maps of different simple mirror twin varieties in Lafayette augite phenocrysts. (**A**) Simple mirror twins parallel to (100). Insets are three-dimensional (3D) representations of the twins. (**B**) Complex variety of simple twinning parallel to (100). In all maps, simple and mechanical twins are highlighted by yellow and red lines, respectively.

Grain-relative orientation distribution (GROD) angle maps derived from EBSD measurements of Lafayette and MIL 03346 show that they have two microstructurally distinct regions, hereafter referred to as “pristine” and “deformed.” Within the pristine regions, augite phenocrysts have little internal misorientation (<1° to 2°, blue colors in GROD angle maps). They contain simple “mirror” twins, which are defined by 180° rotation around the <001> axis forming a twin plane parallel to (100) (Figs. 1 to 3 and figs. S3 and S4). In most cases, twins bisect the phenocrysts (Fig. 3A) (*3*). However, locally, complex mirror twins occur, whereby the central portion of the crystal contains two mirror twins (Fig. 3B).

**Fig. 4:**
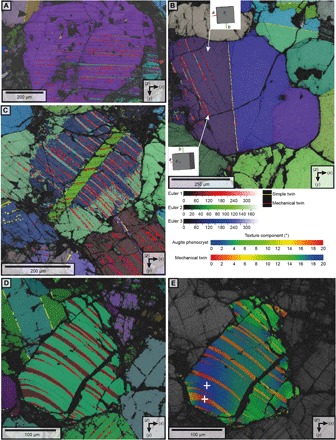
Microstructural characteristics of deformed areas including shock-related mechanical twinning, continuous crystal lattice bending, and high frequency of fractures. High-resolution (0.5- to 1.5-μm step size) EBSD maps of Lafayette. (**A**) Euler map of a typical mechanical twin parallel to (001). The inset shows the 3D representation of the twins. (**B**) Euler map of chevron-like mechanical twinning parallel to (001) developed in an augite phenocryst with preexisting mirror twinning. (**C**) Euler map of mechanical twins that are discontinuous/displaced by fractures. (**D**) Euler image of bent mechanical twins. (**E**) Texture component map of the same area imaged in (D) indicating that the deformation in both the twin and the crystal is related to continuous crystal plastic deformation. The colors represent increasing degrees of misorientation away from the + symbol. Simple twins and mechanical twins are highlighted by yellow and red lines, respectively.

The deformed regions are 1 to 3 mm in size. They are characterized by a high density of crystals with an internal misorientation of >2° (depicted in the GROD angle maps as green, yellow, and red colors with increasing internal misorientation) and are closely spatially associated with areas of high mesostasis abundance (Figs. 1 and 2). Augite phenocrysts exhibit substantial levels of internal misorientation (up to 10°), and most of them contain mechanical twins (Figs. 1, 2, and 4). Misorientation, which is generally concentrated at contact points between the phenocrysts, is particularly apparent in contacts aligned parallel to the trend of the deformed regions (Figs. 1 and 2). Mechanical twins are generally straight and crosscut the internal misorientations (Figs. 1 and 2 and figs. S3 and S4). They occur as thin (0.5 to 3 μm) repeating lamellae defined by a 180° rotation around the <100> axis, forming twin planes parallel to (001) (Fig. 4A). Within individual crystals, these twins vary in orientation across mirror twin planes to produce a chevron pattern (Fig. 4B). In most cases, only one side of a simple twinned crystal shows mechanical twins (Fig. 4B). Furthermore, high-resolution EBSD maps of mechanically twinned crystals reveal that these twins are displaced (by 1 to 10 μm) by fractures that crosscut augite phenocrysts (Fig. 4C). In a few cases, the mechanical twins are bent and tapered (Fig. 4, D and E). This bending of twin boundaries is associated with systematic distortion of the crystal lattices of both the host crystal and the twins (Fig. 4, D and E). The regions of intense deformation are also associated with lower-quality (i.e., lower contrast) electron backscatter patterns (EBSPs) than the undeformed regions (Figs. 1 and 2). The deformed regions lie on the plane of the <c> axis girdle distribution reported in the same meteorites by Daly *et al.* (*4*), which was interpreted as a foliation derived from a gravity-driven crystal settling in the parent magmatic body.

### Mesostasis aqueous alteration assemblages

**Fig. 5:**
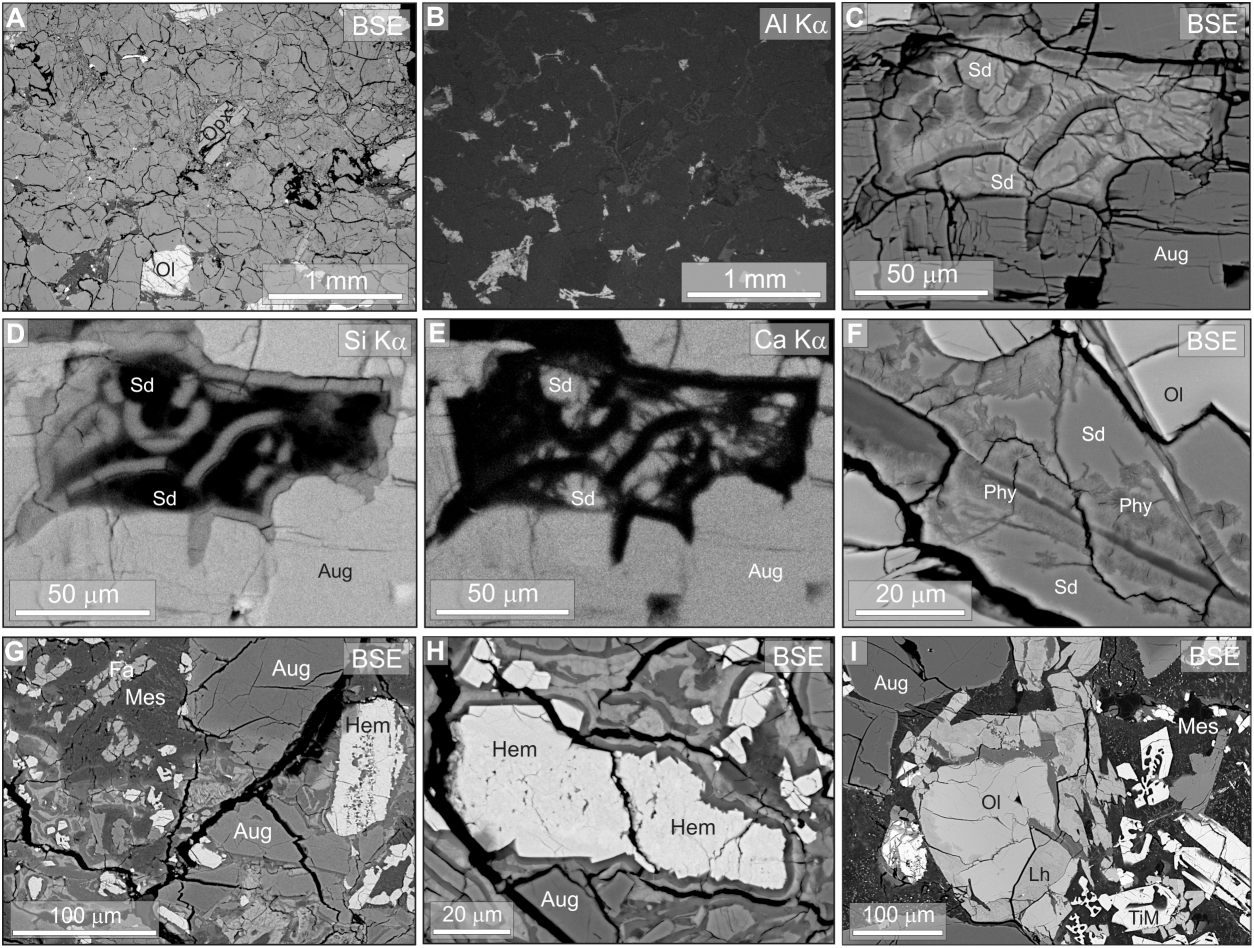
The nature and distribution of mesostasis alteration within Lafayette and MIL 03346 meteorites. BSE and EDS images of mesostasis alteration in Lafayette (A to F) and MIL 03346 (G to I). (**A**) BSE image of an area that contains both pristine and altered mesostasis. Augite is mid-gray, olivine (Ol) is white, orthopyroxene (Opx) is light gray, and pristine mesostasis is dark gray. (**B**) Al Kα x-ray map of (A). Areas of pristine mesostasis are white as they have retained Al. Mesostasis in the middle and upper right of the field of view has been altered with loss of Al. (**C**) BSE image of an area of mesostasis surrounded by augite (Aug) that has been completely altered to siderite (Sd) and phyllosilicate. (**D**) Si Kα x-ray map of (C). Phyllosilicate is light gray, and siderite (Sd) is black. (**E**) Ca Kα x-ray map of (C). Siderite (Sd) is light gray, and phyllosilicate is black. (**F**) A representative vein of alteration products in olivine (Ol) comprising siderite (Sd) that is crosscut by bands and rosettes of phyllosilicate (Phy). (**G**) An area containing pristine mesostasis (Mes) with fayalite laths (Fa) that is juxtaposed with altered mesostasis containing hematite (Hem, white), the low-*Z* phase (dark gray), and high-*Z* phase (light gray). Grains of augite (Aug) are unaltered. (**H**) An area of altered mesostasis containing grains of hematite (Hem, white) that are rimmed by bands of the low-*Z* phase (dark gray) and high-*Z* phase (light gray). (**I**) An area of pristine mesostasis (Mes) containing glass (dark gray) and titanomagnetite (TiM) crystals (white). Unaltered grains of augite (Aug) and an olivine (Ol) grain that is host to several veins of iddingsite (mid-gray) are also shown. Parts of the olivine grain that have a lower *Z* may be laihunite (Lh).

The mesostasis within deformed regions of both meteorites has in places been altered to a suite of secondary phases (Figs. 1, 2, and 5); these phases differ in mineralogy and chemical composition between Lafayette and MIL 03346 (described below; Table 1). In between deformed regions, the mesostasis is pristine, i.e., containing feldspar, fayalite, titanomagnetite, cristobalite, sulfides, and apatite (Figs. 1, 2, and 5).

**Table 1:** Chemical compositions of the alteration assemblages in MIL 03346 in weight % (wt %) ± 1 SD.

	**Olivine-hosted** **iddingsite**	**Mesostasis alteration products**		
		**Low-*Z***	**High-*Z***	**Hematite**
Na_2_O	0.17 ± 0.06	0.38 ± 0.24	0.43 ± 0.20	0.06 ± 0.08
MgO	3.26 ± 0.14	3.27 ± 0.40	0.83 ± 0.70	0.11 ± 0.09
Al_2_O_3_	1.35 ± 0.56	3.50 ± 0.59	1.29 ± 0.66	0.91 ± 0.25
SiO_2_	45.53 ± 1.27	46.09 ± 1.43	20.06 ± 5.12	1.02 ± 0.98
S	0.23 ± 0.06	0.28 ± 0.07	1.14 ± 0.49	0.03 ± 0.13
K_2_O	0.08 ± 0.07	0.28 ± 0.21	0.05 ± 0.07	0.01 ± 0.04
CaO	0.12 ± 0.10	0.16 ± 0.24	0.57 ± 0.43	0.24 ± 0.24
TiO_2_	0.07 ± 0.07	0.04 ± 0.07	0.04 ± 0.07	3.01 ± 1.38
MnO	0.54 ± 0.08	0.40 ± 0.07	0.81 ± 0.67	0.07 ± 0.13
FeO	32.63 ± 1.08	30.11 ± 1.45	53.89 ± 2.48	87.13 ± 2.16*
Total	83.98 ± 1.56	84.51 ± 1.65	79.11 ± 4.18	92.59 ± 0.70
*n*	17	29	18	41

*96.81 wt % as Fe_2_O_3_, giving a total of 102.28 wt %.

### Lafayette

Altered areas of Lafayette mesostasis, highlighted in x-ray maps by Al depletion (Fig. 5, A and B), contain siderite and phyllosilicate (Fig. 5, C to E). Siderite grains are a few tens of micrometers in size and have a composition of Ca_0.33_Mg_0.01_Fe_0.59_Mn_0.07_CO_3_ (*15*). Phyllosilicates mostly occur as ~4-μm-wide selvages between siderite and enclosing augite grains but also crosscut the siderite as veins, curved bands, and radial-fibrous rosettes (Fig. 5, C to E). Olivine grains throughout the thin section contain siderite and phyllosilicate with comparable microstructures to the mesostasis alteration products (Fig. 5F). Siderite within olivine (Ca_0.30_Mg_0.00_Fe_0.45_Mn_0.23_CO_3_) is richer in Mn than in the mesostasis. Phyllosilicates have a comparable Fe-rich smectite chemistry in both contexts (*15*), although there are some mineralogical differences [i.e., Fe-serpentine in the mesostasis and ferric saponite within olivine (*19*)].

### MIL 03346

Mesostasis within the MIL 03346 deformation bands contains ~20- to 150-μm-size grains of Ti-bearing hematite along with two fine-grained Si- and Fe-rich phases that differ in their Si/Fe ratio and mean atomic number (*Z*) (hereafter referred to as “low-*Z*” and “high-*Z*” phases) (Fig. 5G and Table 1). Hematite has previously been identified in the mesostasis of MIL 03346 (*16*, *17*). The Si- and Fe-rich phases occur as “patches” up to a few tens of micrometers in size and as narrow (~1 to 2 μm thick), concentric layers coating hematite and augite (Fig. 5H). The concentric layers have a consistent textural paragenesis whereby the low-*Z* phase is in contact with the hematite or augite (Fig. 5H). The chemical composition of the high-*Z* phase is inconsistent with a single mineral. Its low analytical totals demonstrate the presence of unanalyzed OH/H_2_O, which, together with high-Fe content, suggests that the high-*Z* phase contains ferrihydrite or goethite [which where pure have 85 and 90 weight % (wt %) Fe_2_O_3_, respectively], probably with absorbed Si. Grains of olivine, which mostly occur outside of the deformation bands, contain iddingsite veins (Fig. 5I). Olivine-hosted iddingsite is similar in chemical composition to the mesostasis low-*Z* phase apart from having slightly lower concentrations of Si, Al, and alkali elements and higher concentrations of Fe (Table 1). High-resolution transmission electron microscopy images reveal that iddingsite in olivine from MIL 090032 (a paired stone of MIL 03346) comprises nanocrystalline Fe-smectite (*20*), and so the mesostasis low-*Z* phase probably has a similar mineralogy.

### Numerical modeling

Numerical modeling was performed to investigate the local stress distribution and location of shock-induced damage to be expected within a nakhlite-like material that has been subject to shock. These models assume that the material behaves linearly elastically under maximum tensile or maximum compressive stress. When a microstructure that resembles Lafayette in terms of crystal-mesostasis distribution is shocked to 10 GPa, local stresses reach >15 GPa and are concentrated in areas with abundant crystal-mesostasis interfaces (Fig. 2). Consequently, these are the areas in which the most damage and deformation twinning is expected. These results match our observations. Furthermore, numerical simulations show that when the nakhlite foliation [Fig. 2; (*4*)] is perpendicular or up to 45° to the first principal stress direction, σ_1,_ the damaged region is concentrated along the foliation and in areas with abundant crystal-mesostasis interfaces. When σ_1_ is parallel to the foliation, damage is more uniformly distributed throughout the rock texture (fig. S1).

## DISCUSSION

The close spatial correlation between areas of deformation and aqueous alteration (Figs. 1, 2, and 5) suggests a causal relationship between the two processes: i.e., deformation facilitated ingress of water and its interaction with the mesostasis. A key question then arises: What was the relative timing of deformation and aqueous alteration? Four scenarios require consideration: (i) Deformation was linked to magmatism (~1.4 to 1.3 Ga ago), which was followed by aqueous alteration that was initiated by a separate event ~633 Ma ago; (ii) shock before ~633 Ma ago deformed the nakhlites, and aqueous alteration occurred in response to a separate event ~633 Ma ago; (iii) alteration was initiated by an event ~633 Ma ago, and the shock effects observed are due to the nakhlite ejection impact ~11 Ma ago; and (iv) shock ~633 Ma ago deformed the nakhlites and generated heat to drive a hydrothermal system that was responsible for aqueous alteration. Below, we discuss the relative merits of each scenario and argue that our petrographic, mineralogical, crystallographic evidence and numerical simulations support the model of linked shock deformation and aqueous alteration.

### Origin of the deformation microstructures

The deformed regions in Lafayette and MIL 03346 (Figs. 1 and 2) are petrographically similar to areas of brecciation described in the nakhlites Nakhla and Northwest Africa 998 (*1*, *21*). Thus, this deformation process has affected meteorites sourced throughout the nakhlite lava pile. We first discuss the origin of these microstructures, specifically whether they could have formed during (i) eruption and cooling of these rocks or (ii) a postmagmatic impact.

The deformation of augite phenocrysts (Figs. 1 to 4) is characterized by continuous crystal lattice bending occurring throughout whole grains, while distinct subgrain boundaries are absent. These microstructures show that deformation was not associated with recovery or shear [e.g., (*22*)]. Recovery in pyroxene is a high-temperature (>700°C) process, occurring during crystal plastic deformation (*23*) and/or postdeformation annealing (*22*) and/or magmatic shear. Thus, deformation of the augite phenocrysts cannot have taken place during high-temperature magmatic flow and/or postdeformational annealing. Instead, deformation must have been related to low-temperature crystal plasticity because under these conditions, defects cannot be rearranged into low-energy, low-angle boundaries (*24*). For silicate minerals such as olivine and pyroxene, low-temperature plasticity occurs at ~10 to 15% of the melting temperature and high pressure (*24*); hence, the temperatures associated with this type of deformation would be expected to be ~200°C (*24*).

The observed brittle deformation must also have occurred after the lava had crystallized; otherwise, fractures formed while magma was available would have been infilled with mesostasis, which is not observed. Fractures could still have formed during the interval between when the rock was fully crystallized and when the lava reached ambient temperature (~0°C); however, because fractures crosscut twins and alteration (*1*), this possibility is unlikely.

Furthermore, if deformation was associated with magmatism and/or magmatic flow, it would be expected to concentrate in regions of high phenocryst abundances due to stresses associated with compaction. However, our numerical models and petrographic observations indicate that the opposite is true, and stress and strain are concentrated in areas of higher mesostasis abundances.

Last, mechanical augite twins with clear (100)-parallel twin boundaries are absent from our samples. These twins form in magmatic systems that are being deformed during cooling (*25*), at low temperatures and under stresses of 140 to 150 MPa (*26*). Shear stresses produced in terrestrial lava flows can be quite high [1000 MPa (*27*)]. The fact that these twins are absent suggests that the foliation defined by crystal alignment formed during gravity settling during emplacement. In such a scenario, only low stresses would be expected to occur between settling grains, especially given the relatively low gravity of Mars (*4*). Consequently, our observations indicate that deformation was not magmatic and therefore must be from a postmagmatic event.

The tapered, (001)-parallel mechanical twins in Lafayette and MIL 03346 augite phenocrysts are diagnostic of shock (*1*, *3*, *8*, *9*). The observed presence/absence of mechanical twins across some mirror twin boundaries (Fig. 4C) is typical for elastic anisotropy of minerals in different crystal orientations (*28*). These mechanical twins can be reconciled with low levels (5 to 15 GPa) of shock metamorphism (*9*). The fact that both the twin boundaries and the internal crystal lattice of some mechanical twins are bent constrains the timing of twinning (Fig. 4, D and E). Twins form straight facets with an internally undeformed structure; therefore, these twins are likely to have formed before crystal plastic deformation. Consequently, the temporal relationships between crystal plasticity and twinning, as well as the low-temperature nature of crystal plastic deformation, further constrain the formation of these two features as being related to the same low-temperature shock metamorphic event.

The colocation of grains with internal crystal plastic deformation and shock twins within deformed regions of Lafayette and MIL 00346 indicates a common cause, interpreted to be shock metamorphism (Figs. 1 and 2 and figs. S3 and S4) (*1*, *3*, *8*, *9*). The heterogeneous distribution of the deformed regions is typical for the passage of a shock wave through a material with an inherent anisotropy (*29*). Daly *et al.* (*4*) showed that Lafayette and MIL 03346 exhibit both a planar shape–preferred orientation and a planar crystallographic–preferred orientation, and these petrofabrics were interpreted to have originated from gravity settling of augite crystals during magmatic emplacement. The regions of deformation lie in the same orientation as the magmatic flow foliation (Figs. 1 and 2). Note that the flow foliation is not only defined by shape alignment of the phenocrysts but also the heterogeneous distribution of mesostasis in parallel with the shape-preferred orientation. Our numerical simulations for rocks with similar mesostasis-phenocryst distributions as Lafayette indicate that stress spikes of up to 1.5 times the bulk shock pressure are expected in regions of high mesostasis-phenocryst abundance (Fig. 2 and fig. S1). This coincidence of predicted high-stress areas suggests that the rheological anisotropy provided by the preferred alignment of phenocrysts and correlated variations in phenocryst abundance had a direct influence on the loci of shock-related damage and deformation. The fractures that crosscut twins are interpreted to have formed from the decompression tail following the compressional shock wave (*30*). Consequently, these later deformation microstructures postdate foliation development and their association with other shock related features described above strongly suggests that they constitute another signature of shock metamorphism.

In summary, the sequence of deformation events interpreted from microstructural characteristics and numerical simulations is as follows: (i) Magmatic flow aligned phenocrysts to produce a foliation and heterogeneous distribution of crystals and mesostasis; in combination, these two properties gave the nakhlites a substantial rheological anisotropy (*4*); (ii) shock metamorphism produced mechanical twins, followed by low-temperature plastic deformation within grains and brecciation by fracturing within distinct damage zones. This rheological anisotropy localized stresses to produce the regions of concentrated shock deformation.

### Aqueous alteration of the nakhlites

Olivine grains throughout the Lafayette and MIL 00346 thin sections have been aqueously altered to form iddingsite veins, thus demonstrating that liquid water penetrated throughout both rocks. The more severe aqueous alteration of olivine in comparison to augite reflects the considerably greater reactivity of olivine in the presence of Martian fluids (*31*). The mesostasis of Lafayette and MIL 03346 is pristine except in regions containing shock-deformed augite grains where it has been aqueously altered (Figs. 1 and 2). The mineralogical, compositional, and microstructural similarities between olivine- and mesostasis-hosted alteration products suggest that they formed from the same generation of aqueous solutions and therefore formed at the same time (i.e., ~633 Ma). However, fluid chemistry would have differed between the mesostasis and olivine if a substantial proportion of the solutes had been sourced from the dissolution of these components. This local derivation of ions is highlighted in MIL 03346 by small differences in chemical composition, particularly Al and Fe, between the mesostasis low-*Z* alteration phase and olivine-hosted iddingsite (Table 1). Siderite in Lafayette also differs in its Fe/Mn ratio between the mesostasis (Ca_0.33_Mg_0.01_Fe_0.59_Mn_0.07_CO_3_) and olivine (Ca_0.30_Mg_0.00_Fe_0.45_Mn_0.23_CO_3_) (*15*). Together, these observations indicate that water-rock ratios were low.

### Relative timing of shock metamorphism and alteration: A model for nakhlite postemplacement history

The focusing of alteration in the deformed regions of Lafayette and MIL 03346 suggests that shock substantially increased the subsequent reactivity of the mesostasis in the presence of liquid water. One mechanism could be that locally high concentrations of fractures and brecciation served to enhance the porosity, permeability, and surface area/volume ratio of the mesostasis. Alternatively, maskelynite may have also formed within deformed regions of the mesostasis, further enhancing their susceptibility to aqueous alteration (*32*). The production of maskelynite from mesostasis feldspar should start at >15 GPa (*8*, *9*) and would be complete at shock pressures of 35 GPa (*9*). Although previous estimates place shock metamorphism in nakhlites below the maskelynite stability field [i.e., 5 to 15 GPa (*33*)], the temperatures required for low-temperature plasticity in pyroxene are ~200°C, which is 100°C higher than expected for a 5 to 15 GPa shock event (*9*, *24*, *33*). To attain temperatures of ~200°C, a localized shock pressure of ~30 GPa (*9*, *33*) is required. It is well known that shock pressures are highly heterogeneous when passing through mineralogically heterogeneous materials or those with a petrofabric [e.g., (*29*)]. In the case of Lafayette and MIL 03346, heterogeneity was provided by the magmatic foliation fabric, the porphyritic texture, and the nonuniform concentration of mesostasis and phenocrysts (*1*, *2*, *4*). Our numerical simulations support this idea in demonstrating that pressure spikes of 1.5 times the average shock stress were concentrated around areas of high mesostasis abundance (Fig. 2). Thus, these relatively mild impacts could locally generate the shock pressures and postshock temperatures required for maskelynite formation. Therefore, our microstructural data suggest that it may have been possible to form maskelynite in discrete parts of Lafayette and MIL 03346. Although maskelynite has not been described from the nakhlites, there have been reports of shock melts in Nakhla that support our numerical model and further demonstrate localized compressional and tensional stresses within and above the maskelynite stability field (*34*).

The petrographic evidence that mesostasis alteration is focused in deformed regions suggests that an impact predated aqueous alteration. Therefore, we constrain the timing of shock deformation to the ~700 Ma between cooling of the igneous rocks at ~1.4 to 1.3 Ga ago (*5*) and aqueous alteration ~633 Ma ago (*6*). A second impact then ejected these rocks from Mars ~11 Ma ago (*1*). Because the phyllosilicates, carbonates, and oxides show no signs of having been shocked, with only few descriptions of minor fractures crosscutting the iddingsite veins in olivine (*1*), the ~11 Ma old impact must have generated quite low (<5 GPa) pressures, in agreement with Fritz *et al.* (*33*). Our numerical simulations place an additional constraint on the position of the second impact in relation to the first. Our model suggests that, where σ1 is parallel to the magmatic foliation, the deformation is homogenously distributed (fig. S1). Conversely, where σ1 is normal to or up to 45° to the foliation, the resultant deformation is heterogeneously distributed and matches the pattern in Lafayette and MIL 00346 (Fig. 2). If the foliation present in these meteorites was gravity driven, then the plane of foliation would have been approximately parallel to the Martian surface (*4*). Thus, the second impact site can be constrained to having been located such that the propagating shock wave enters perpendicular to the foliation. This geometry can be found either directly below the ~633 Ma old crater as the shock wave propagates downward (i.e., the ~11 Ma old crater was within the older crater) or distal to the ~633 Ma old crater as the propagating shock wave curves back up toward the surface. The ~11 Ma old impact site is unlikely to be within the first crater because, in such a location, highly shocked lithologies and potentially even impact melt sheets would be expected to occur stratigraphically above the mildly shocked nakhlites, yet no such lithologies with ~11 Ma old ejection ages have been documented in the Martian meteorite record. Therefore, the ~11 Ma old crater is likely to have formed on the area affected by the earlier crater.

We have no empirical constraints on the timing of the first impact. However, because our microstructural and modeling results show that high-shock temperatures (locally ~200°C) were generated within the nakhlites, this impact could have provided sufficient heat to drive aqueous alteration (*10*), thus implying a date of ~633 Ma ago. If shock and aqueous alteration were not linked, a third impact or magmatic/fluvial activity ~633 Ma ago would have to be postulated as a driver of aqueous alteration. While another impact ~633 Ma ago is possible, it would need to have been more energetic than the pre-633 Ma old event to have driven aqueous alteration, yet there is no evidence for the nakhlites having been shocked three times. The possibility of magmatic activity ~633 Ma ago cannot be discounted but could only be proven if a Martian meteorite were found that had a cosmogenic exposure age of ~11 Ma ago and a crystallization age of ~633 Ma old. This may still be the case because the nakhlite sample set is small (20 rocks), and so younger extrusive/intrusive rocks may still be found. Fluvial activity ~633 Ma ago may have been a source of water, but the temperature of these fluids would be too low (approximately ambient) to drive alteration of olivine (>150°C) (*35*). We therefore conclude that nakhlite aqueous alteration was driven by an impact ~633 Ma ago that generated sufficient heat to melt subsurface ices and drive a hydrothermal cell that, size dependent, could have been quite long lived (*7*).

Our results predict that the nakhlite launch site hosts two spatially related craters, one that formed ~633 Ma ago and the other ~11 Ma ago. The initial impact to generate shock metamorphism and an associated hydrothermal cell could have left a crater as small as 2 km in diameter (*10*). Ejecting material from Mars requires at least a 200-m bolide resulting in a 3-km crater (*36*). There are only ~53 young (~11 Ma old) rayed craters on lithologically and temporally (~1 Ga old) compatible surfaces on Mars that are potential candidates for the nakhlite launch site (*37*). Some of these candidate craters are situated near older craters. However, it may not be possible to positively identify any single crater as the launch site because some potential candidate craters may have been obscured and so overlooked because of erosive processes (*37*) and the ejection event may well have produced a later, larger crater that could have completely overprinted the ~633 Ma old structure.

## CONCLUSIONS

Our petrographic, mineralogical, and microstructural results, supported by numerical modeling, provide compelling evidence for two impacts at the nakhlite launch site. An impact ~633 Ma ago generated most of the shock microstructures in Lafayette, MIL 03346, and several of the other nakhlites. Shock-induced stresses and temperatures in localized regions reached ~30 GPa and no less than 200°C, accounting for crystal plastic deformation and twinning of augite. The ~633 Ma old impact also provided the heat source for the hydrothermal system that drove aqueous alteration, which focused on mesostasis in the deformed regions owing to its higher porosity and permeability, and the likely presence of maskelynite. An impact ~11 Ma ago ejected the nakhlites from Mars resulting in only mild microstructural deformation including fracturing. Together, our results constrain the nakhlite ejection site to contain two adjacent impact craters: one that formed ~633 Ma ago and the other ~11 Ma ago.

## MATERIALS AND METHODS

MIL 03346 was found in Antarctica in 2003. It has mass of 715.2 g and a weathering grade B and is paired with MIL 090030, MIL 090032, and MIL 090136. This study used one thin section of MIL 03346 (118). This study also used one thin section of Lafayette, USNM 1505-5, an 800-g fall or very fresh find, recognized in the collection of Purdue University in the United States in 1931.

For SEM imaging and energy dispersive x-ray spectroscopy (EDS) measurements at the University of Glasgow, thin sections of the nakhlite’s MIL 03346 (section 118) and Lafayette (section USNM 1505-5) were coated with 20 nm of carbon. Backscattered electron (BSE) images and quantitative x-ray analyses were obtained from the thin sections after carbon coating, using a Zeiss Sigma VP field-emission gun (VP-FEGSEM) operated at 20 kV/2 nA and high vacuum. For each analysis, the electron beam was rastered over an area of ~9 μm^2^ to minimize volatile loss, and x-rays were collected for 60 s, using an Oxford Instruments 80-mm^2^ X-Max silicon drift detector energy-dispersive spectrometer. Spectra were processed using Oxford Instruments INCA software and quantified using the following standards: Na (jadeite), Mg (periclase), Al (corundum), Si and Ca (diopside), P (apatite), S (pyrite), K (K-feldspar), Ti (rutile), Cr (chromite), Mn (rhodonite), and Fe (garnet). Typical detection limits (in wt % oxide) are as follows: Na_2_O (0.10), MgO (0.07), Al_2_O_3_ (0.11), SiO_2_ (0.11), CaO (0.12), S (0.08), K_2_O (0.09), TiO_2_ (0.10), Cr_2_O_3_ (0.28), MnO (0.22), and FeO (0.23). The modal mineral abundances for MIL 03346 (118) were calculated by point counting in the BSE image by overlaying a 32 × 46 grid of equally spaced points. Resin points were not counted, giving a total number of 1212 measurements.

To prepare the samples for EBSD analysis, the carbon coat was removed with ethanol. The Lafayette and MIL 03346 sections were mechanically polished using a glycol suspension of 1-μm aluminum spheres, followed by a glycol suspension of 0.3-μm aluminum spheres (5 min each) and last, using a NaOH colloidal silica suspension for 30 min and 4 hours, respectively, to remove the damage layer of the initial mechanical polish and to provide a suitable surface for EBSD analyses.

EBSD data were acquired at the University of Glasgow using a Zeiss Sigma VP-FEGSEM operated in variable pressure (49 Pa) mode to negate the need to apply a carbon coat and give an enhanced signal. For EBSD data acquisition, each sample was tilted to 70°, the standard angle used for EBSD data collection. Simultaneous EDS and EBSD data were collected using an Oxford Instruments X-Max 80-mm^2^ silicon drift detector energy dispersive spectrometer and an Oxford Instruments NordlysMax^2^ EBSD detector, respectively. EBSD data were collected using an accelerating voltage of 20 kV and the automated Large Area Mapping module in Oxford Instruments Aztec 3.3 software. The area analyzed by EBSD was 106.4 mm^2^ for MIL 03346 and 85 mm^2^ for Lafayette using a step size of 4 μm, producing a total of 6.6 and 5.3 million EBSPs, respectively. In addition, regions of interest associated with crystal deformation were targeted in each section for higher-resolution EBSD mapping using a lower step size of 1.5 μm.

EBSPs were analyzed in Oxford Instruments Aztec 3.3 software using a 4 × 4 binning and an exposure time of 32.1 ms for MIL 03346 and 40 ms for Lafayette. The EBSPs for both sections were collected with a frame average of 1 to facilitate rapid mapping of most of the section. The mean angular deviation, an assessment of the quality of the pattern indexing where <1 is considered good, was 0.52 and 0.61 for augite in MIL 03346 and Lafayette, respectively.

The EBSD data were noise-reduced using Oxford Instruments HKL software Channel 5 by a wild-spike correction that removes isolated data points followed by iterative eight- and seven-point nearest-neighbor zero solutions and a single six-point nearest-neighbor zero solution. This procedure aids in defining grains without creating substantial artifacts (*38*). Grain boundaries within the dataset were defined by <10° misorientation across adjacent pixels. The internal deformation within each grain was evaluated using a GROD angle map. Here, the average orientation of a grain is defined and compared to all other data points within the same grain. The data points are color-coded to reflect the variation in misorientation (0 to 10°) of each point relative to the average grain orientation.

To model the damage distribution that results from the passage of a shockwave through a rock with a foliation, and variable distribution of mesostasis and phenocrysts as observed in the nakhlite meteorites, we used a linear elastic model using a COMSOL Multiphysics software, which is based on a finite element method. To focus on the microscale, boundary effects were eliminated by defining a sufficiently large geometry (14 mm by 21 mm) and using a mixed mesh method. The latter method allows a large geometry to be modeled at low resolution where regions of complicated, intricate microstructures (e.g., high differences in elastic constants and irregular geometries) are modeled at high resolution. By placing the regions of interests far from the outer boundaries of the model, boundary effects are minimized. We used a triangular mesh for both crystals and areas of mesostasis to aid in the stability and accuracy of the simulation. The mesh resolution was 529,754 elements where most of the elements were concentrated in the region of interest (fig. S2). The mesostasis and phenocrysts within the nakhlites have substantially different elastic constants where augite is substantially harder [bulk modulus = 129.5 × 10^9^ N/m^2^, shear modulus = 81.1 × 10^9^ N/m^2^, and density = 3221 kg/m^3^ (*39*)] than basaltic glass [bulk modulus = 62.9 × 10^9^ N/m^2^, shear modulus = 36.5 × 10^9^ N/m^2^, and density = 2777 kg/m^3^ (*40*)] that we take to represent the mesostasis. The investigated microstructures, e.g., distribution of mesostasis and phenocrysts, were chosen to represent that of the mesostasis-phenocryst distribution observed in the part of Lafayette (cf. yellow box in Fig. 2C). The model was run oriented with σ1 at 0°, 45°, and 90° to the orientation of the foliation (see fig. S2) (*4*). All meshes were also exposed to a tensile σ3 at 0°, 45°, and 90° to the orientation of the foliation. This sequence of compressive and then tensile stresses was chosen to model the passage of a 10 GPa shock wave. As a result, it is possible to monitor the loci of stresses produced during shock metamorphism. The result of these models maps out regions of the model where irreversible damage would be expected to occur.

## Supplementary Material

http://advances.sciencemag.org/cgi/content/full/5/9/eaaw5549/DC1

Download PDF

Boom boom pow: Shock-facilitated aqueous alteration and evidence for two shock events in the Martian nakhlite meteorites

## SUPPLEMENTARY MATERIALS

Supplementary material for this article is available at http://advances.sciencemag.org/cgi/content/full/5/9/eaaw5549/DC1 Fig. S1. Figures of the numerical impact model run at different angles of the principal stress axis and the anisotropy of the microstructures, i.e., the foliation and mesostasis-phenocryst distribution. Fig. S2. Representation of the numerical mesh used for the simulations shown. Fig. S3. High-resolution inverse pole figure map of MIL 03346 highlighting the distribution of twinned augite crystals. Fig. S4. High-resolution inverse pole figure map of Lafayette highlighting the distribution of twinned augite crystals.

## References

[1] Treiman A. H., The nakhlite meteorites: Augite-rich igneous rocks from Mars. Chem. Erde Geochem. 65, 203–270 (2005).

[2] Bunch T. E., Reid A. M., The nakhlites Part I: Petrography and mineral chemistry. Meteorit. Planet. Sci. 10, 303–315 (1975).

[3] J. Berkley, K. Keil, M. Prinz, in *Lunar and Planetary Science Conference Proceedings* (1980), vol. 11, pp. 1089–1102.

[4] Daly L., Piazolo S., Lee M. R., Griffin S., Chung P., Campanale F., Cohen B. E., Hallis L. J., Trimby P. W., Baumgartner R., Forman L. V., Benedix G. K., Understanding the emplacement of Martian volcanic rocks using petrofabrics of the nakhlite meteorites. Earth Planet. Sci. Lett. 520, 220–230 (2019).

[5] Cohen B., Mark D. F., Cassata W. S., Lee M. R., Tomkinson T., Smith C. L., Taking the pulse of Mars via dating of a plume-fed volcano. Nat. Commun. 8, 640 (2017). 28974682 10.1038/s41467-017-00513-8 PMC5626741

[6] Borg L., Drake M. J., A review of meteorite evidence for the timing of magmatism and of surface or near-surface liquid water on Mars. J. Geophys. Res. Planets 110, 10.1029/2005JE002402, (2005).

[7] Hallis L., Taylor G. J., Nagashima K., Huss G. R., Needham A. W., Grady M. M., Franchi I. A., Hydrogen isotope analyses of alteration phases in the nakhlite martian meteorites. Geochim. Cosmochim. Acta 97, 105–119 (2012).

[8] Greshake A., Fritz J., Stöffler D., Petrology and shock metamorphism of the olivine-phyric shergottite Yamato 980459: Evidence for a two-stage cooling and a single-stage ejection history. Geochim. Cosmochim. Acta 68, 2359–2377 (2004).

[9] Stöffler D., Keil K., Scott E. R. D., Shock metamorphism of ordinary chondrites. Geochim. Cosmochim. Acta 55, 3845–3867 (1991).

[10] Osinski G. R., Tornabene L. L., Banerjee N. R., Cockell C. S., Flemming R., Izawa M. R. M., McCutcheon J., Parnell J., Preston L. J., Pickersgill A. E., Pontefract A., Sapers H. M., Southam G., Impact-generated hydrothermal systems on Earth and Mars. Icarus 224, 347–363 (2013).

[11] Corrigan C. M., Velbel M. A., Vicenzi E. P., Modal abundances of pyroxene, olivine, and mesostasis in nakhlites: Heterogeneity, variation, and implications for nakhlite emplacement. Meteorit. Planet. Sci. 50, 1497–1511 (2015).

[12] Udry A., Day J. M. D., 1.34 billion-year-old magmatism on Mars evaluated from the co-genetic nakhlite and chassignite meteorites. Geochim. Cosmochim. Acta 238, 292–315 (2018).

[13] Lee M., Tomkinson T., Hallis L., Mark D., Formation of iddingsite veins in the martian crust by centripetal replacement of olivine: Evidence from the nakhlite meteorite Lafayette. Geochim. Cosmochim. Acta 154, 49–65 (2015).

[14] Harvey R. P., McSween H. Y. Jr., Petrogenesis of the nakhlite meteorites: Evidence from cumulate mineral zoning. Geochim. Cosmochim. Acta 56, 1655–1663 (1992).

[15] Tomkinson T., Lee M. R., Mark D. F., Smith C. L., Sequestration of Martian CO_2_ by mineral carbonation. Nat. Commun. 4, 2662 (2013). 24149494 10.1038/ncomms3662 PMC4354006

[16] Day J. M. D., Taylor L. A., Floss C., McSween H. Y. Jr., Petrology and chemistry of MIL 03346 and its significance in understanding the petrogenesis of nakhlites on Mars. Meteorit. Planet. Sci. 41, 581–606 (2006).

[17] Ling Z., Wang A., Spatial distributions of secondary minerals in the Martian meteorite MIL 03346, 168 determined by Raman spectroscopic imaging. J. Geophys. Res. Planets 120, 1141–1159 (2015).

[18] Hallis L. J., Taylor G. J., Comparisons of the four Miller Range nakhlites, MIL 03346, 090030, 090032 and 090136: Textural and compositional observations of primary and secondary mineral assemblages. Meteorit. Planet. Sci. 46, 1787–1803 (2011).

[19] Hicks L. J., Bridges J. C., Gurman S., Ferric saponite and serpentine in the nakhlite martian meteorites. Geochim. Cosmochim. Acta 136, 194–210 (2014).

[20] Hallis L. J., Ishii H. A., Bradley J. P., Taylor G. J., Transmission electron microscope analyses of alteration phases in Martian meteorite MIL 090032. Geochim. Cosmochim. Acta 134, 275–288 (2014).

[21] Treiman A. H., Irving A. J., Petrology of martian meteorite Northwest Africa 998. Meteorit. Planet. Sci. 43, 829–854 (2008).

[22] Song R., Ponge D., Raabe D., Kaspar R., Microstructure and crystallographic texture of an ultrafine grained C–Mn steel and their evolution during warm deformation and annealing. Acta Mater. 53, 845–858 (2005).

[23] Urai J. L., Means W. D., Lister G. S., Dynamic recrystallization of minerals. Miner. Rock Deform. Lab. Stud. 36, 161–199 (1986).

[24] Trepmann C. A., Renner J., Druiventak A., Experimental deformation and recrystallization of olivine–processes and timescales of damage healing during postseismic relaxation at mantle depths. Solid Earth 4, 423–450 (2013).

[25] Trepmann C., Stöckhert B., Mechanical twinning of jadeite–an indication of synseismic loading beneath the brittle–plastic transition. Int. J. Earth Sci. 90, 4–13 (2001).

[26] Orzol J., Trepmann C. A., Stöckhert B., Shi G., Critical shear stress for mechanical twinning of jadeite—An experimental study. Tectonophysics 372, 135–145 (2003).

[27] Deubelbeiss Y., Kaus B. J. P., Connolly J. A. D., Caricchi L., Potential causes for the non-Newtonian rheology of crystal-bearing magmas. Geochem. Geophys. Geosyst. 12, Q05007 (2011).

[28] Huffman A. R., Reimold W. U., Experimental constraints on shock-induced microstructures in naturally deformed silicates. Tectonophysics 256, 165–217 (1996).

[29] Bland P. A., Collins G. S., Davison T. M., Abreu N. M., Ciesla F. J., Muxworthy A. R., Moore J., Pressure–temperature evolution of primordial solar system solids during impact-induced compaction. Nat. Commun. 5, 5451 (2014). 25465283 10.1038/ncomms6451 PMC4268713

[30] H. J. Melosh, The contact and compression stage of impact cratering, in *Impact Cratering: Processes and Products* (2012), pp.32–42.

[31] Lee M. R., Tomkinson T., Mark D. F., Stuart F. M., Smith C. L., Evidence for silicate dissolution on Mars from the Nakhla meteorite. Meteorit. Planet. Sci. 48, 224–240 (2013).

[32] Hallis L. J., Kemppinen L., Lee M. R., Taylor L. A., The origin of alteration “orangettes” in Dhofar 019: Implications for the age and aqueous history of the shergottites. Meteorit. Planet. Sci. 52, 2695–2706 (2017).

[33] Fritz J., Artemieva N., Greshake A., Ejection of Martian meteorites. Meteorit. Planet. Sci. 40, 1393–1411 (2005).

[34] Malavergne V., Guyot F., Benzerara K., Martinez I., Description of new shock-induced phases in the Shergotty, Zagami, Nakhla and Chassigny meteorites. Meteorit. Planet. Sci. 36, 1297–1305 (2001).

[35] Changela H. G., Bridges J. C., Alteration assemblages in the nakhlites: Variation with depth on Mars. Meteorit. Planet. Sci. 45, 1847–1867 (2010).

[36] Head J. N., Melosh H. J., Ivanov B. A., Martian meteorite launch: High-speed ejecta from small craters. Science 298, 1752–1756 (2002). 12424385 10.1126/science.1077483

[37] Kereszturi A., Chatzitheodoridis E., Searching for the source crater of nakhlite meteorites. Orig. Life Evol. Biosph. 46, 455–471 (2016). 27021613 10.1007/s11084-016-9498-x

[38] Bestmann M., Prior D. J., Intragranular dynamic recrystallization in naturally deformed calcite marble: Diffusion accommodated grain boundary sliding as a result of subgrain rotation recrystallization. J. Struct. Geol. 25, 1597–1613 (2003).

[39] R. F. S. Hearmon, The elastic constants of crystals and other anisotropic materials. In: *Landolt-Börnstein Tables III*, K.H. Hellwegge, A.M. Hellwegge, eds. (Springer-Verlag, Berlin, 1984), pg. 559.

[40] Meister R., Robertson E. C., Werre R. W., Raspet R., Elastic moduli of rock glasses under pressure to 8 kilobars and geophysical implications. J. Geophys. Res. Solid Earth 85, 6461–6470 (1980).

