# Supplementary material for: Boom boom pow: Shock-facilitated aqueous alteration and evidence for two shock events in the Martian nakhlite meteorites
Source: Sci Adv. 2019 Sep 4;5(9):eaaw5549. doi: 10.1126/sciadv.aaw5549 (PMC6726442; doi:10.1126/sciadv.aaw5549)
Supplement: http://advances.sciencemag.org/cgi/content/full/5/9/eaaw5549/DC1 [file supp_5_9_eaaw5549__index.html]

Science Advances | Science AdvancesAAASSearchScience AdvancesMenu

## Supplementary Materials

**This PDF file includes:**

- Fig. S1. Figures of the numerical impact model run at different angles of the principal stress axis and the anisotropy of the microstructures, i.e., the foliation and mesostasis-phenocryst distribution.
- Fig. S2. Representation of the numerical mesh used for the simulations shown.
- Fig. S3. High-resolution inverse pole figure map of MIL 03346 highlighting the distribution of twinned augite crystals.
- Fig. S4. High-resolution inverse pole figure map of Lafayette highlighting the distribution of twinned augite crystals.

Download PDF

**Files in this Data Supplement:**

- Adobe PDF - aaw5549\_SM.pdf
