# Supplementary material for: Boom boom pow: Shock-facilitated aqueous alteration and evidence for two shock events in the Martian nakhlite meteorites
Source: Sci Adv. 2019 Sep 4;5(9):eaaw5549. doi: 10.1126/sciadv.aaw5549 (PMC6726442; doi:10.1126/sciadv.aaw5549)
Supplement: Download PDF [file aaw5549_SM.pdf]

Science Advances
AAAS

advances.sciencemag.org/cgi/content/full/5/9/eaaw5549/DC1

Supplementary Materials for

Boom boom pow: Shock-facilitated aqueous alteration and evidence for two shock events in the Martian nakhlite meteorites

L. Daly*, M. R. Lee, S. Piazolo, S. Griffin, M. Bazargan, F. Campanale, P. Chung, B. E. Cohen, A. E. Pickersgill, L. J. Hallis, P. W. Trimby, R. Baumgartner, L. V. Forman, G. K. Benedix

*Corresponding author. Email: luke.daly@glasgow.ac.uk

Published 4 September 2019, *Sci. Adv.* **5**, eaaw5549 (2019)
DOI: 10.1126/sciadv.aaw5549

This PDF file includes:

Fig. S1. Figures of the numerical impact model run at different angles of the principal stress axis and the anisotropy of the microstructures, i.e., the foliation and mesostasis-phenocryst distribution.
Fig. S2. Representation of the numerical mesh used for the simulations shown.
Fig. S3. High-resolution inverse pole figure map of MIL 03346 highlighting the distribution of twinned augite crystals.
Fig. S4. High-resolution inverse pole figure map of Lafayette highlighting the distribution of twinned augite crystals.

Supplementary Materials

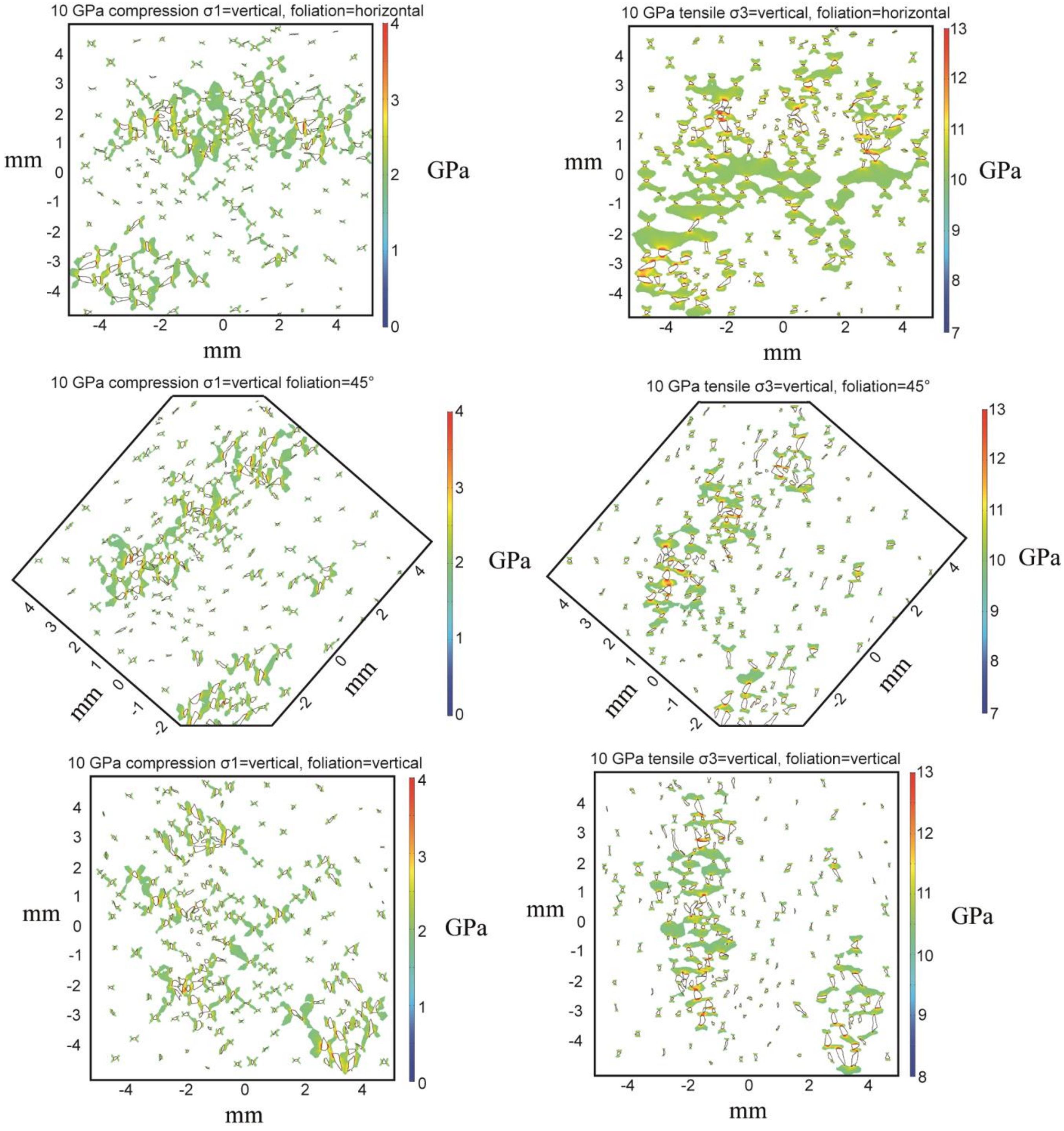

Fig. S1. Figures of the numerical impact model run at different angles of the principal stress axis and the anisotropy of the microstructures, i.e., the foliation and mesostasis-phenocryst distribution.

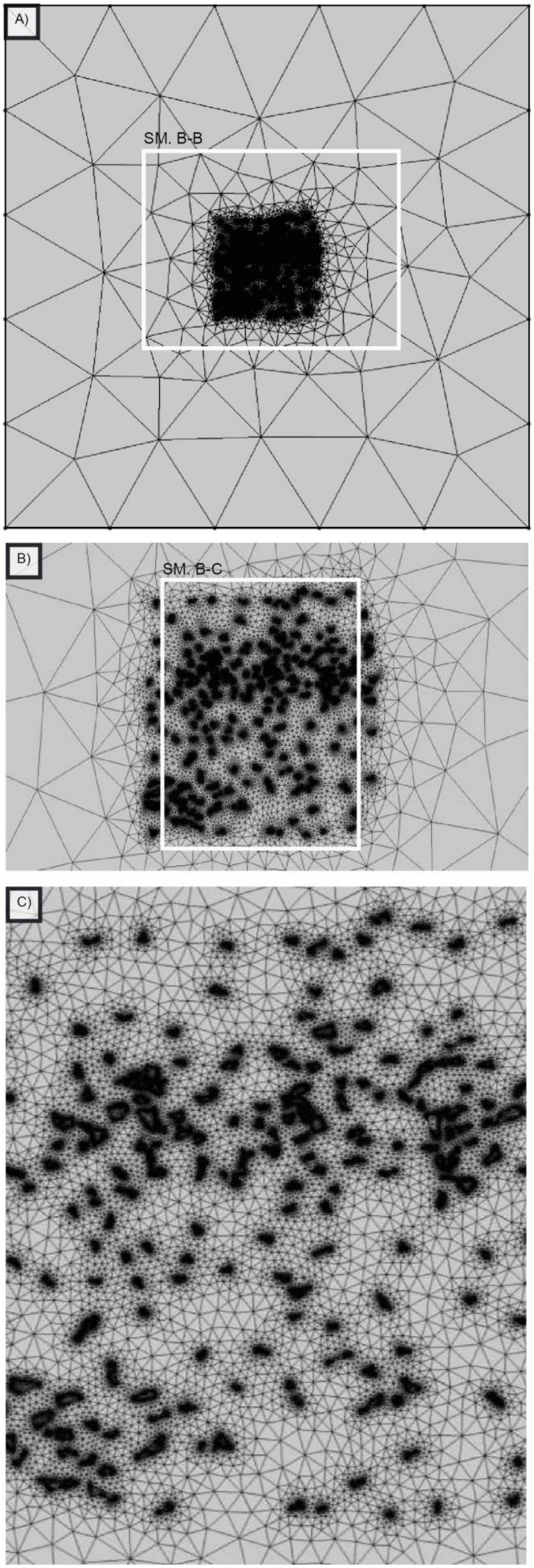

A)
SM. B-B
B)
SM. B-C
C)

Fig. S2. Representation of the numerical mesh used for the simulations shown. Note that the model is in fact much larger than the area of interest. This is to minimize boundary effects. The flexible meshing ensures high accuracy and stability in areas of high microstructural, hence elastic, heterogeneity.

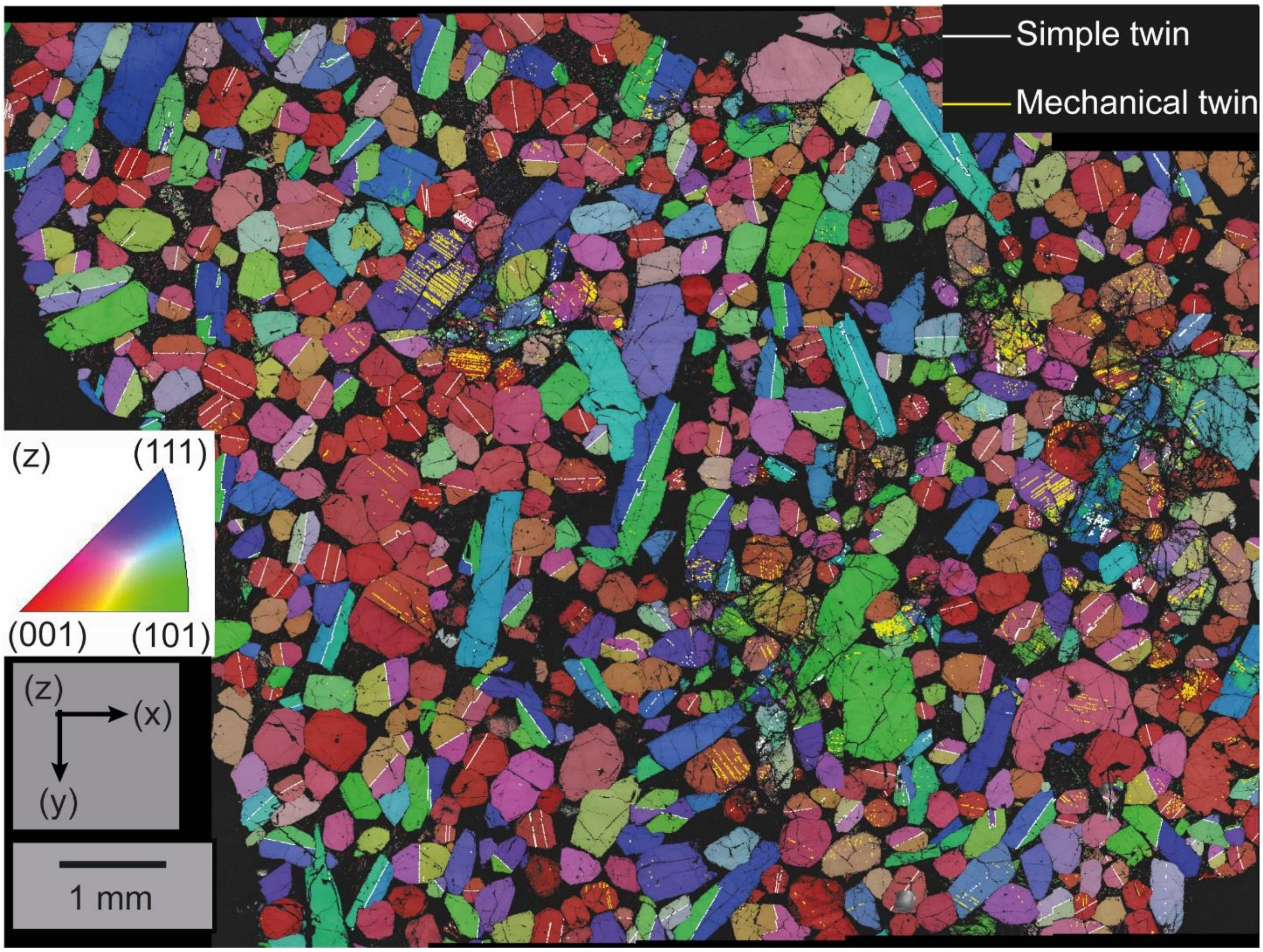

Fig. S3. High-resolution inverse pole figure map of MIL 03346 highlighting the distribution of twinned augite crystals. Mechanical twins, whose boundaries are shown as yellow lines occur exclusively within the deformed areas of Fig. 1B**.** Simple twin boundaries are shown as white lines and occur throughout the sample.

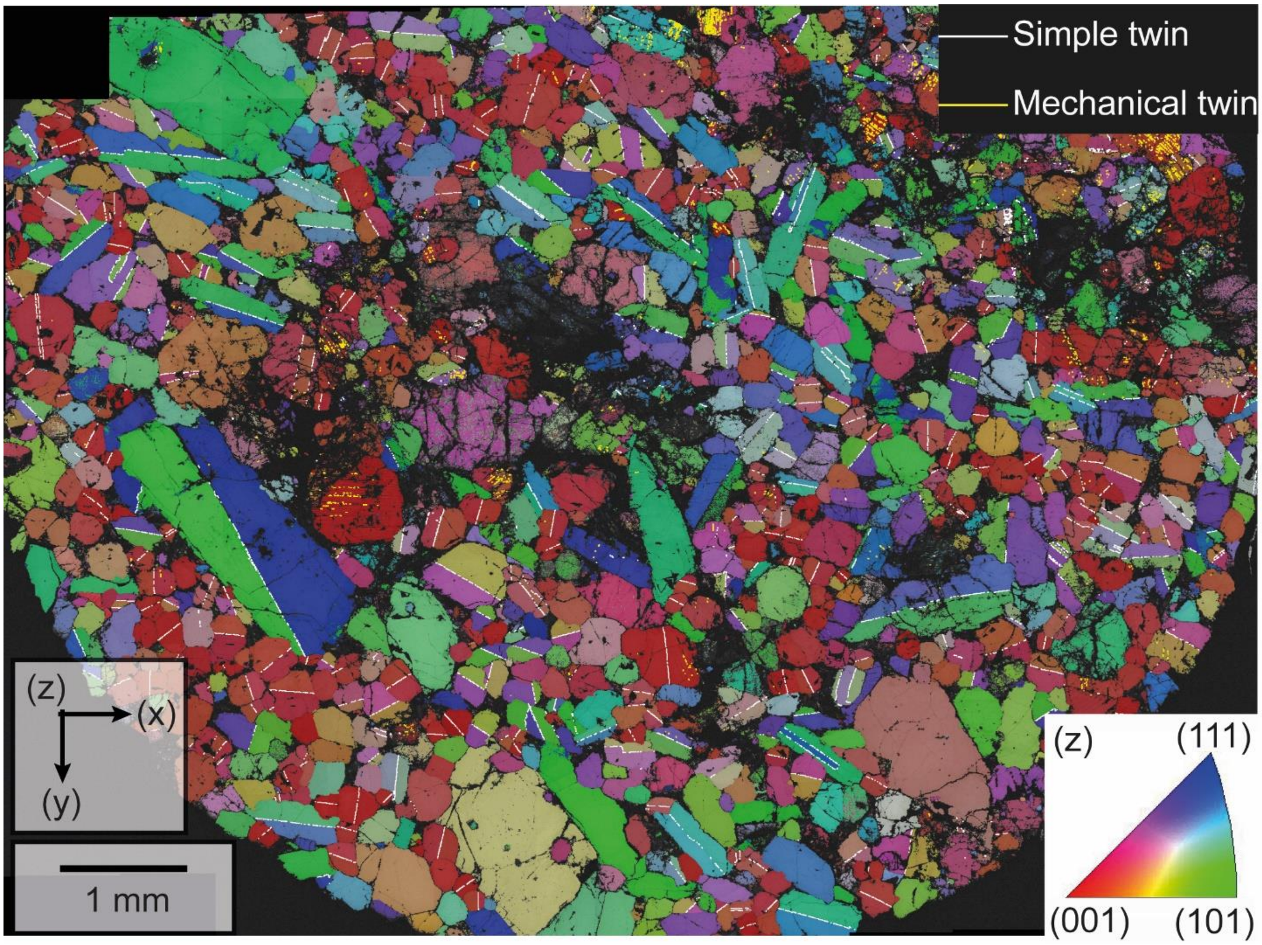

Fig. S4. High-resolution inverse pole figure map of Lafayette highlighting the distribution of twinned augite crystals. Mechanical twins, whose boundaries are shown as yellow lines occur exclusively within the deformed areas of Fig. 1B**.** Simple twin boundaries are shown as white lines and occur throughout the sample.

Supplementary Materials

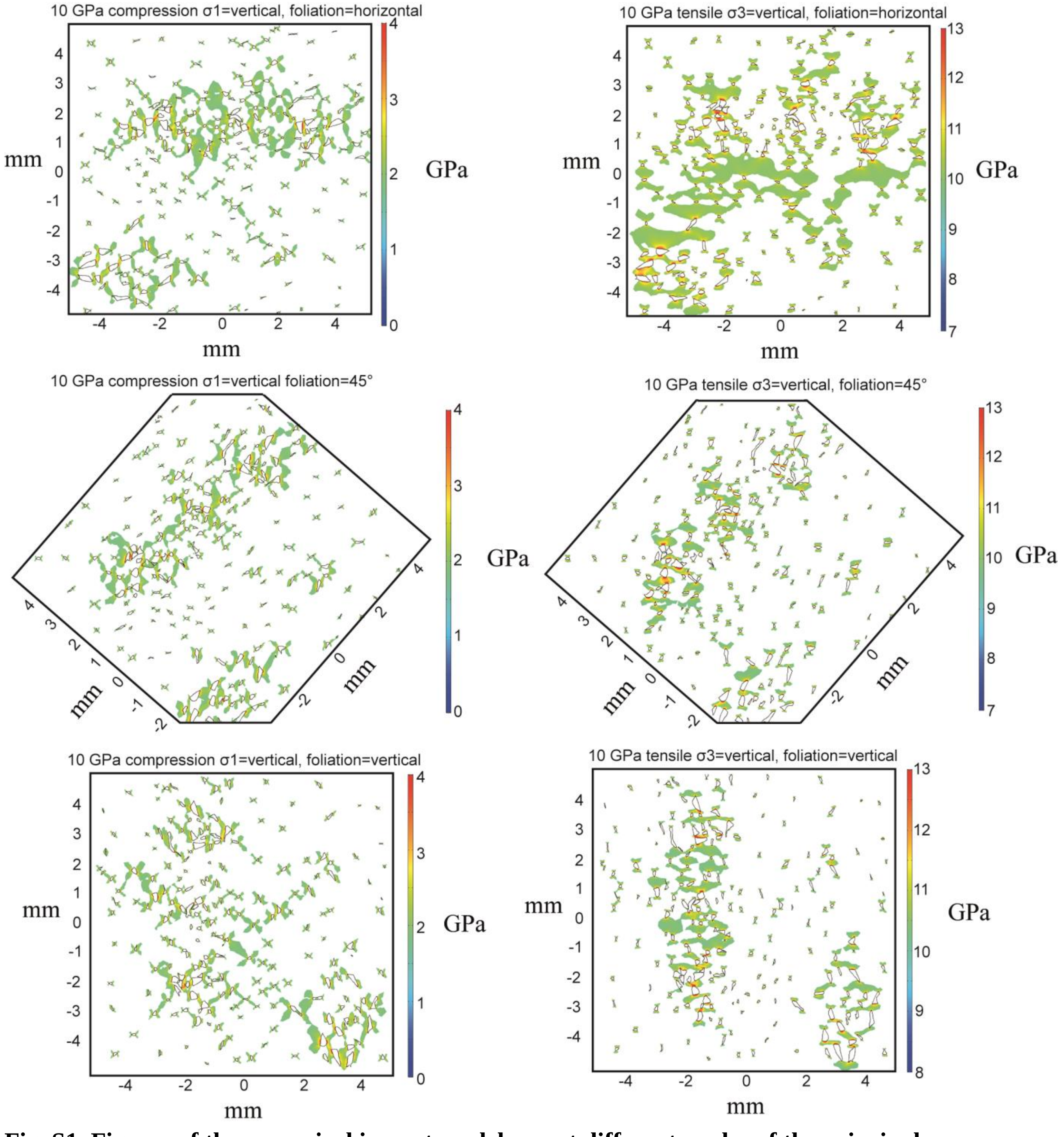

Fig. S1. Figures of the numerical impact model run at different angles of the principal stress axis and the anisotropy of the microstructures, i.e., the foliation and mesostasis-phenocryst distribution.

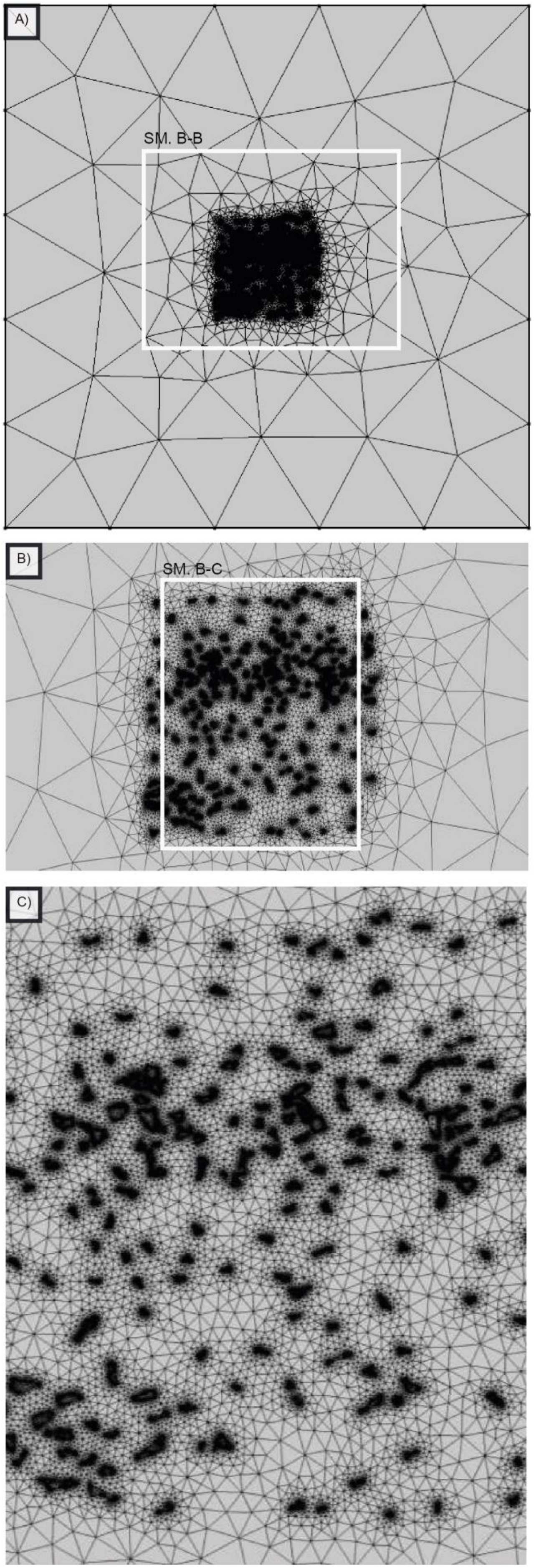
A)
SM. B-B
B)
SM. B-C
C)

Fig. S2. Representation of the numerical mesh used for the simulations shown. Note that the model is in fact much larger than the area of interest. This is to minimize boundary effects. The flexible meshing ensures high accuracy and stability in areas of high microstructural, hence elastic, heterogeneity.

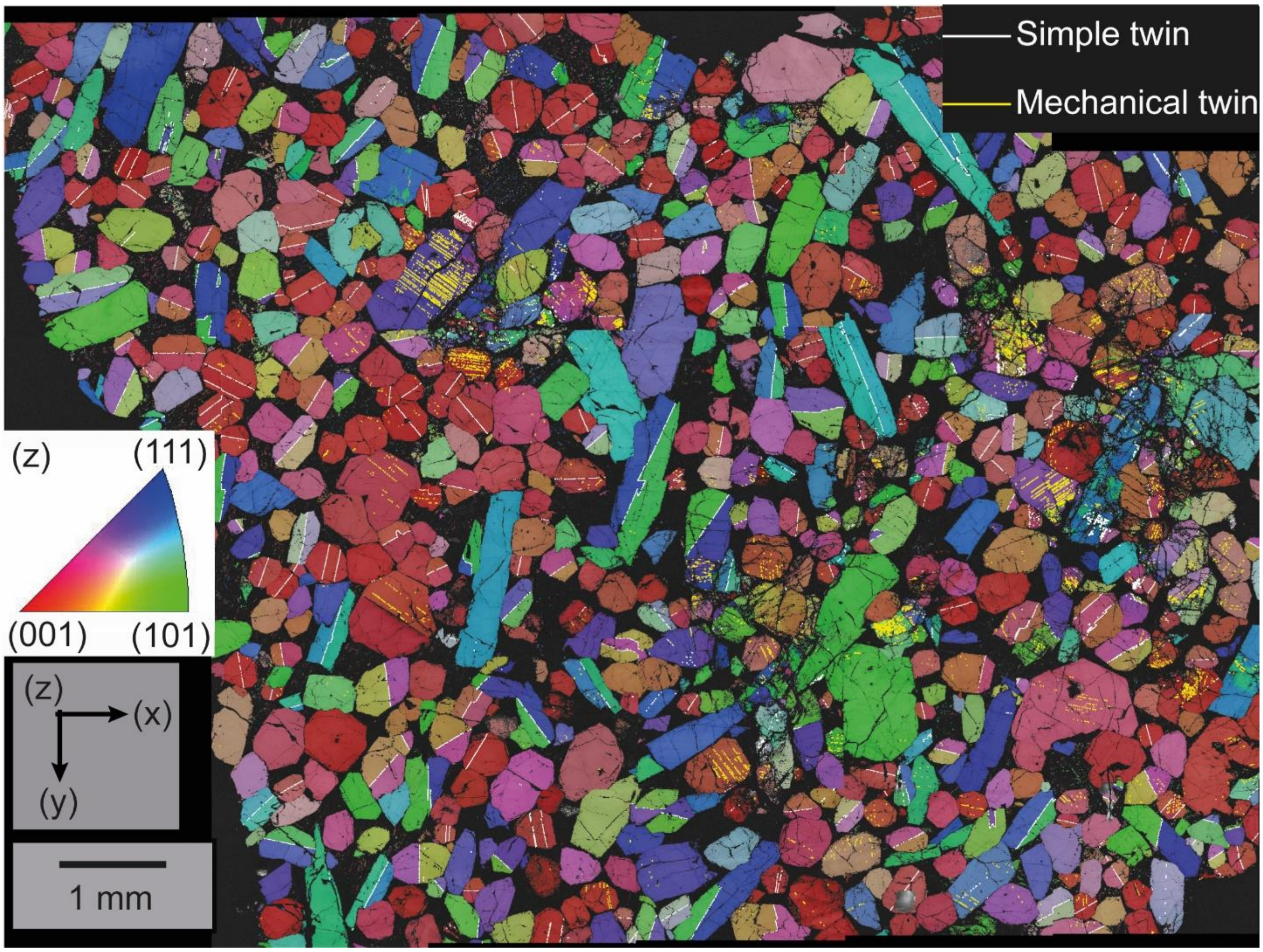

Fig. S3. High-resolution inverse pole figure map of MIL 03346 highlighting the distribution of twinned augite crystals. Mechanical twins, whose boundaries are shown as yellow lines occur exclusively within the deformed areas of Fig. 1B**.** Simple twin boundaries are shown as white lines and occur throughout the sample.

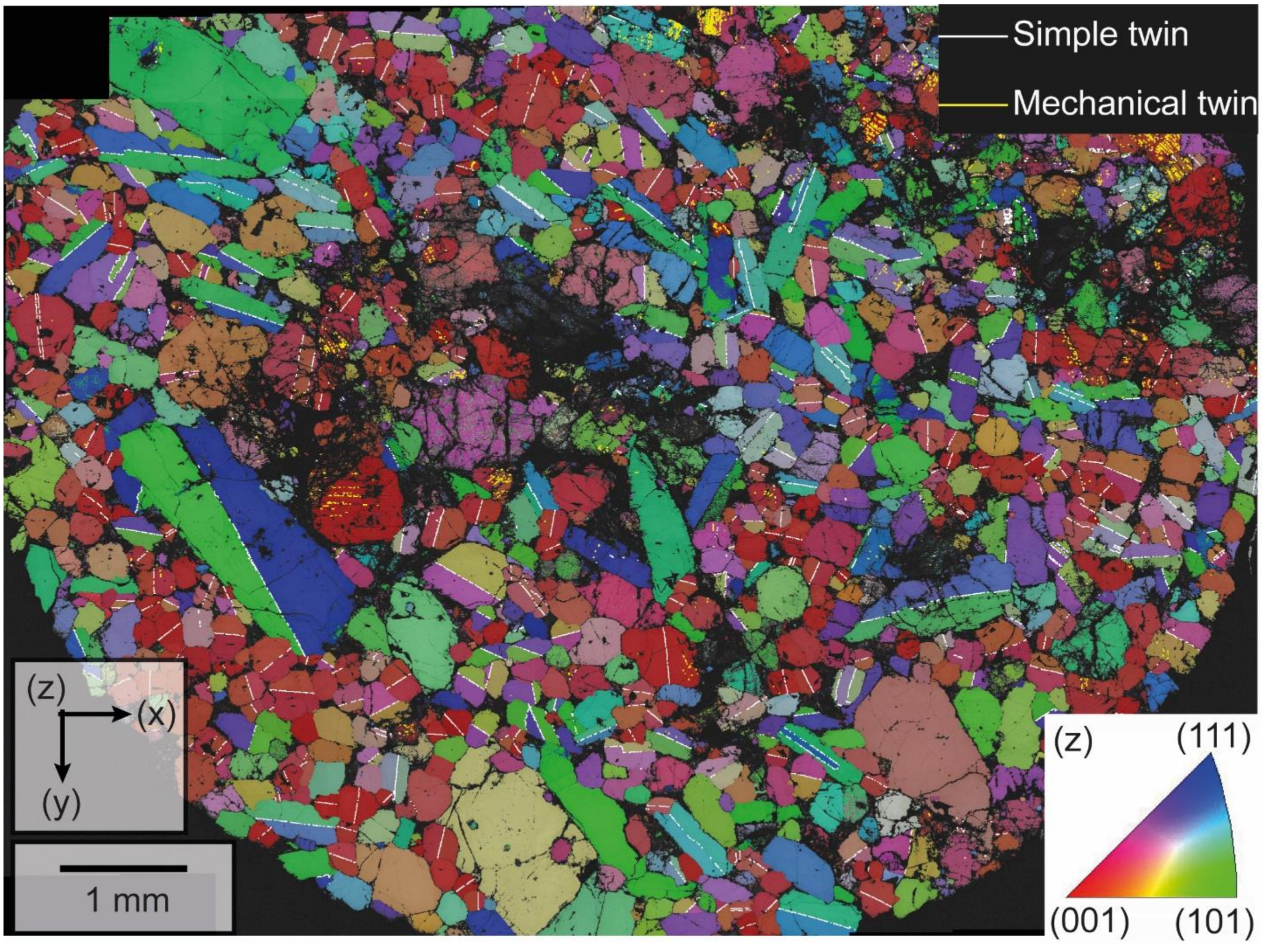

Fig. S4. High-resolution inverse pole figure map of Lafayette highlighting the distribution of twinned augite crystals. Mechanical twins, whose boundaries are shown as yellow lines occur exclusively within the deformed areas of Fig. 1B**.** Simple twin boundaries are shown as white lines and occur throughout the sample.
